# Case report: Rare myeloid sarcoma development following renal transplantation with KRAS and DNMT3A gene mutations

**DOI:** 10.1186/s13000-021-01141-z

**Published:** 2021-08-31

**Authors:** Danyang Wu, Xiaoxuan Lu, Xiaojing Yan, Ran Gao

**Affiliations:** Department of Hematology, the First Affiliated Hospital of China Medical University, No.155, Nanjing North Street, Shenyang, Liaoning 110001 PR China

**Keywords:** Myeloid sarcoma, Renal transplantation, KRAS, DNMT3A, Case report

## Abstract

**Background:**

A high incidence of malignant tumors, such as post-transplant lymphoproliferative disorders (PTLD), Kaposi sarcoma, and renal cancer is common in solid organ and bone marrow transplant recipients. However, myeloid sarcoma (MS) after renal transplantation has rarely been reported and the diagnosis is challenging due to its low incidence.

**Case presentation:**

Here, we report a rare case of a 49-year-old man who developed myeloid sarcoma (MS) in the transplanted kidney two years after renal transplantation. Next-generation sequencing (NGS) showed mutations of KRAS and DNMT3A genes in the MS, and no gene mutations in the bone marrow. He presented a normal karyotype of 46, XY. Following treatment with 6 cycles of systemic chemotherapy, the patient was in satisfactory condition with stable serum creatinine (sCr) levels at the 1-year follow-up. In addition, we performed a detailed review with emphasis on the clinical manifestations, and the diagnostic and therapeutic processes of another 7 patients who developed MS following renal transplantation.

**Conclusions:**

Our report illustrates the clinical utility of comprehensive genomic profiling in benefiting the diagnosis of MS, the selection of therapeutic strategy and the determination of whether MS is donor-derived.

**Supplementary Information:**

The online version contains supplementary material available at 10.1186/s13000-021-01141-z.

## Background

An increased risk of secondary malignant tumors threatens the life of solid organ transplant recipients, which is mainly attributed to immune deficiency due to immunosuppressive therapy after transplantation and chronic viral infections [1]. Donor-derived malignancies occur in a small amount of recipients [2]. MS is a rare myeloid neoplasm characterized as an extramedullary soft tissue mass, which can occur as de novo tumor, recurrent acute myeloid leukemia (AML), myeloproliferative neoplasm (MPN), myelodysplastic syndrome (MDS) or MPN/MDS [3]. The most common involved sites of MS include lymph nodes, skin and soft tissues, bone, testes, peritoneum and gastrointestinal tract [4]. MS derived from transplanted kidney has only been reported in few cases. It is challenging to diagnose de novo MS, especially in patients without a history of hematologic malignancy or when MS involves unusual sites.

The mutation spectrum of MS is generally consistent with that in acute myelocytic leukemia (AML) [5]. RAS pathway mutation and DNMT3A are common in myeloid neoplasms, especially in AML [6]. Furthermore, it has been revealed that RTK-RAS pathway mutation is more enriched in MS than AML and may contribute significantly to the pathogenesis of MS [7]. Hence, the application of comprehensive genomic profiling is helpful for the diagnosis of MS and the selection of optimal therapeutic strategies. Here we report 1 case of renal MS post-renal transplantation with KRAS and DNMT3A mutations. We also identified another 7 cases in the literature which highlight that molecular genotyping analyses are helpful to develop target therapeutic strategy and determine whether MS is donor-derived or as a secondary tumor in association with iatrogenic immunosuppression and chronic viral infections.

## Case presentation

The patient was a 49-year-old man with chronic renal insufficiency. He underwent right renal transplantation in the Chinese People's Armed Police Force General Hospital in November 2016. Following transplantation, the patient underwent immunosuppression therapy, which included tacrolimus, prednisone, and myfortic, resulting in the return of normal renal function in the patient.

**Fig. 1:**
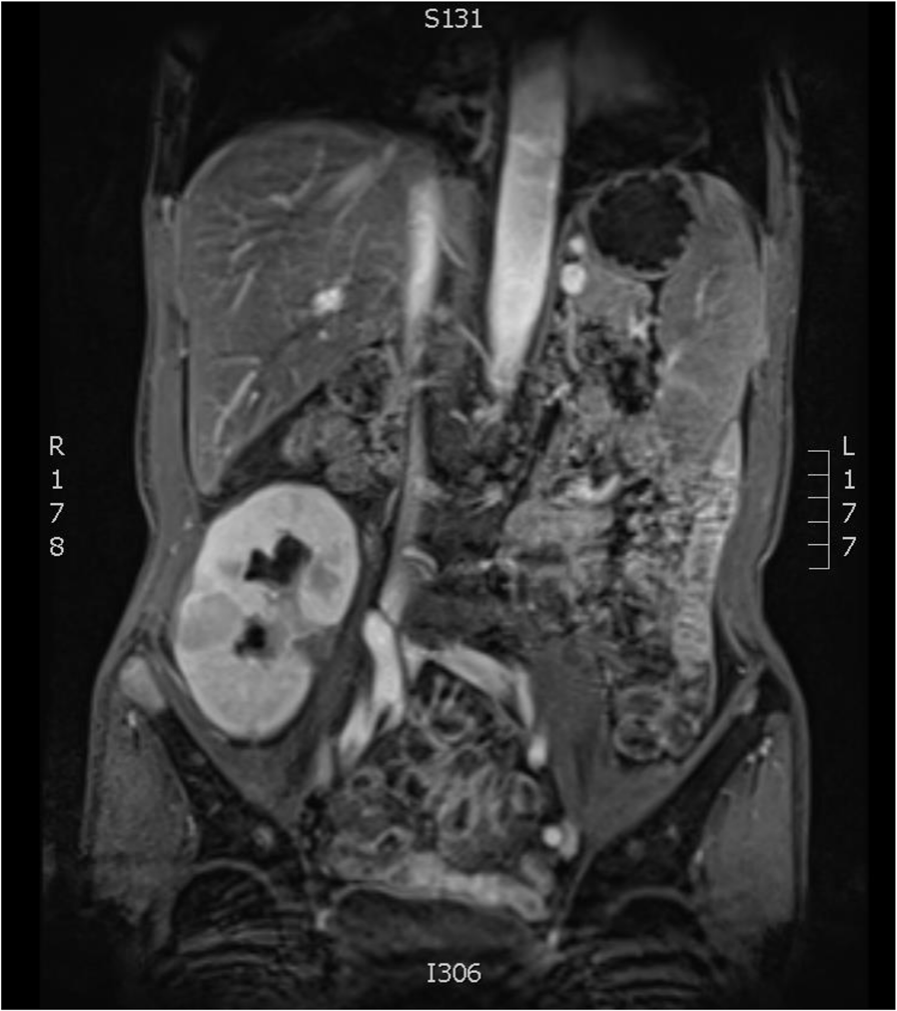
Enhanced MRI demonstrated multiple, variably sized quasi-circular lesions with slightly short signal intensity on T1WI, slightly long signal intensity on T2WI, and high signal intensity on DWI. The larger size lesion was about 4.66 cm by 3.10 cm

Two years later, the level of sCr in the patient gradually increased from normal to 274 mmol/L during routine laboratory testing. Enhanced MRI demonstrated multiple, variably sized quasi-circular lesions with slightly short signal intensity on T1WI, slightly long signal intensity on T2WI, and high signal intensity on DWI. The larger size lesion was about 4.66 cm by 3.10 cm (Fig. 1). PET-CT revealed a soft tissue mass in the pelvis of the transplanted kidney (with an elevated SUV of 2.6), and multiple metastatic hypermetabolic soft tissue density nodules can be observed in the parenchyma of the transplanted kidney (with a maximum SUV of 2.9). No metastatic hypermetabolic lesions were observed in any other parts of the body.

**Fig. 2:**
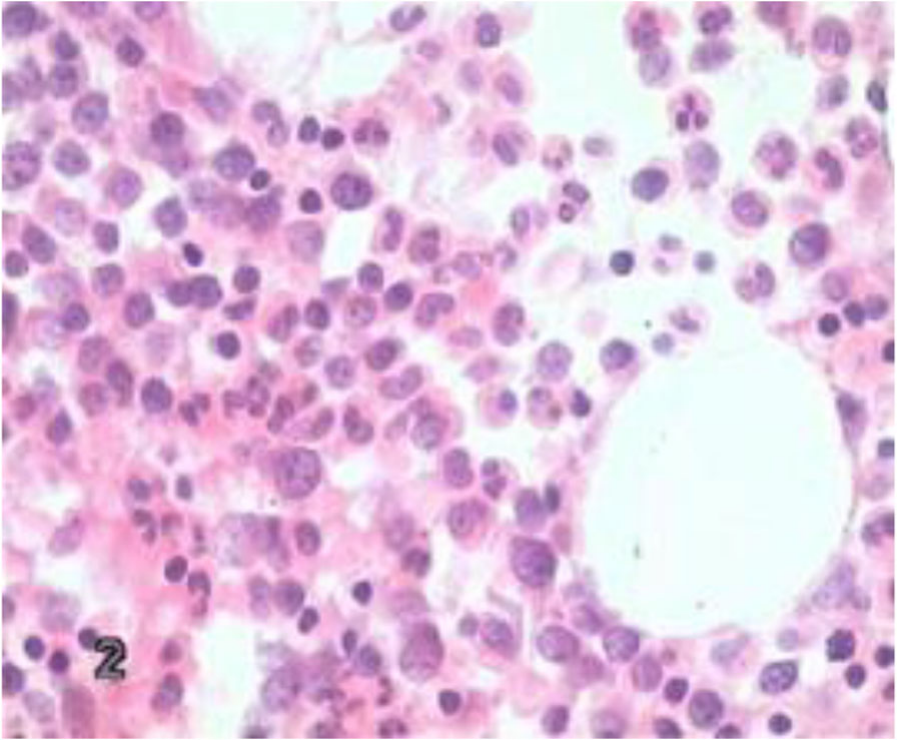
Bone marrow biopsy showed no evidence of leukemic infiltration (original magnification, × 400)

A puncture biopsy of the transplanted kidney was performed, and the results indicated large neoplastic cells. The immunohistochemistry examination revealed the following: CD34 (few +), CD68 (KP1) (few +), CD163 (-), lysozyme (few +), MPO (+), Ki-67 (>70% +), CD3 (few +), CD20 (-), TdT (few +), CD15 (-), CD117 (+), FISH: EBER(-). Bone marrow biopsy showed no evidence of leukemic infiltration (Fig. 2), with normal chromosomes (46, XY). Examinations of fusion genes for both acute lymphoblastic leukemia and myeloid leukemia were negative. NGS examinations of the transplanted kidney was performed on 34 commonly mutated genes in AML (listed in Supplementary Table) and showed mutations of KRAS (NM_004985:exon2:c.G35A:p.G12Drs121913529), DNMT3A (NM_022552:exon15:c.1675delT:p.C559fs) , and no gene mutations in the bone marrow. Eventually, the patient was diagnosed with *de novo* MS in the transplanted kidney. Detailed information on the donor was not available, but the other transplant recipient did not have any problems in the transplanted kidney.

The patient underwent systemic chemotherapy in our hospital in October 2019, and received DA regimen chemotherapy (daunorubicin 100 mg on days 1-3 and cytarabine 170 mg on days 1-7). The patient responded well to the treatment, and the size of the mass in the transplanted kidney decreased and the level of sCr decreased to 192 mmol/L. The patient underwent a second cycle of DA regimen chemotherapy 30 days later. The size of the biggest lesion decreased to 2.5cm by 1.8cm, but the level of sCr remained at around 180 mmol/L. Therefore, we changed the chemotherapy regimen to cytarabine 2.5 g every 12h on days 1, 3, and 5, and etoposide 100 mg on days 1-3 for 3 cycles. However, 3 days after the 5th cycle of the revised chemotherapy regimen, the patient presented with progressive generalized motor weakness and slurred speech, without headache and vomiting, and the patient couldn’t stand and walk without assistance. A routine blood test showed WBC 4×109/L, NEUT 3.84×109/L, HGB 94.3 g/L, and PLT 44×109/L. The patient’s liver function, serum ions, and other metabolic panels were within normal limits, except that the level of sCr was 157 mmol/L. Head CT and MRI did not show any new abnormal acute findings, and it was presumed that the neurologic symptoms experienced by the patient were a result of cytarabine neurotoxicity. Thereafter the patient was treated with prednisone 40 mg twice a day for 3 days, which led to a significant improvement in his symptoms. After recovering from the neurologic symptoms, the patient refused to continue chemotherapy and allogeneic hematopoietic stem cell transplantation. The patient was followed up on April 20, 2021 and he could speak fluently, but sometimes walked with an unsteady step. The size of the biggest lesion in the transplanted kidney decreased to 1.7 cm and the level of sCr was maintained at around 200mol/L.

## Discussion

It is difficult to diagnose *de novo* MS, especially in patients without a history of hematologic malignancy or when MS manifests in unusual sites, so a high index of suspicion is needed. If a diagnosis of MS is suspected, performing immunohistochemistry, flow cytometry, cytogenetic, and molecular studies is critical. From the morphological point of view, MS is easily confused with lymphoma, small round blue cell tumor, and medulloblastoma. So the diagnosis of MS depends mainly on immunohistochemistry examination. The most commonly expressed immunohistochemistry markers include CD68-KP1, myeloperoxidase, CD117, CD99, CD68/PG-M1, lysozyme, CD34, terminal deoxynucleotidyl transferase, CD56, CD61, CD30, glycophorin A, and CD4 [8]. B and T cell markers, such as CD3, CD4, CD20, and CD79a should be examined to differentiate MS from non-Hodgkin lymphomas.

When there is a diagnostic dilemma between MS and other diseases based on morphological and immunhistochemical feature, the application of comprehensive genomic profiling is helpful for the diagnosis of MS, since MS shares the same mutation spectrum as AML. In the case reported herein, the mutations detected in KRAS and DNMT3A, which are considered as important pathogenetic factors in AML, supported the diagnosis of MS.

Furthermore, the clinical use of molecular genotyping analyses is also helpful to guide target therapeutic strategy. We searched MEDLINE/PubMed using the MeSH terms (“kidney transplantation” and “Myeloid Sarcoma”) and (“Myeloid Sarcoma post-renal transplantation”). The search returned 7 cases of MS in post-renal transplantation patients; their clinical manifestations, diagnostic information, and therapeutic processes are presented in Table 1. FISH or molecular genotyping analyses has been systematically applied to the analysis of MS in five patients. Three patients of them were confirmed positive for PML-RARα gene rearrangements through FISH and gained the treatment of all-trans retinoic acid (ATRA) and arsenic trioxide (ATO). So, molecular genotyping analyses are essential to both the diagnosis and the treatment of MS in post-renal transplantation patients.

**Table 1:** Baseline disease characteristics, treatments, and outcomes of MS cases following renal transplantation in the literature review

Reference	Patient 1 [9]	Patient 2 [10]	Patient 3 [10]	Patient 4 [11]	Patient 5 [12]	Patient 6 [13]	Patient 7 [14]
Age/Sex	52/M	72/M	77/F	45/F	26/M	65/M	35/F
Donor	Living unrelated donor	38-year-old female died of brain death	38-year-old female died of brain death	Healthy without any medical history	21-year-old female died of cerebral haemorrhage	54-year-old male died of cerebrovascular accident with PLT 27 × 10^9/L,	21-year-old female died from intracranial hemorrhage with APL
FISH	NA	46,XX donor origin	46,XX donor origin	NA	PML-RARα	PML-RARα t(15;17) gains of 11q and 21q	PML/RAR α
Location	Lateral to the allograft kidney	Allograft kidney	Allograft kidney	Right allograft kidney, skin, breast, left foot	The end of the allograft ureter	Allograft kidney	Allograft kidney
Marrow status	Uninvolved	Uninvolved	Uninvolved	NA	APL	APL	Uninvolved
Treatment	Fludarabine and cytosine arabinoside followed by all-trans retinoic acid and arsenic trioxide	Allograft nephrectomy one cycle of cytarabine and daunorubicin(7 + 3)	Allograft nephrectomy	2 cycles of DA;3 cycles of medium-dose cytarabine; 2 cycles of MAE; Radiotherapy and regular systematic chemotherapy for breast Resecting the lesion in the left foot	Mass resection Radiotherapy 6 cycle of arsenicals plus ATRA	The patient died of cardiac arrest due to coronary artery disease before any treatment was performed for MS	Idarubicin and ATRA following the LPA 99 protocol
Prognosis	Disease free on his last PET-CT 1 year after the initial diagnosis	In remission and on dialysis for another 8 months and died due to cardiovascular disease	In remission and on dialysis for another 18 months and died due to cardiovascular disease.	30 months later, the patient was alive and satisfied	During one year follow-up, urine volume was normal and renal function was stable (sCr 80–100 μmol/L).		ATRA was maintained and complete remission was achieved

The exact mechanism of MS in transplant recipients is still unclear. DNA damage resulting from prolonged exposure to immunosuppressive drug may be one reason [15]. Another risk factor is the chronic viral infection. Donor-derived MS can be either due to direct transmission of malignant hematopoietic stem cells from the donor to the recipient during transplantation, or due to the malignant transformation of donor-derived nonmalignant hematopoietic stem cells in the recipient [16]. For donor-derived MS without systemic involvement, reduction or cessation of immunosuppressive agents and transplant nephrectomy may be sufficient. Therefore, determining whether MS is donor-derived may be helpful for the treatment [10]. There are four patients listed in Table 1 were confirmed that the malignant cells were of donor origin through the use of molecular genotyping analyses.

## Conclusion

In conclusion, we report a rare case of a 49-year-old man with MS in the transplanted kidney and another 7 MS cases post-renal transplantation were reviewed. Although MS rarely occurs in the transplanted kidney, a high index of suspicion is needed. If a diagnosis of MS is suspected, performing immunohistochemistry, flow cytometry, cytogenetic, and molecular studies is critical for confirming the diagnosis. Concurrently, comprehensive genomic profiling is also helpful to determine whether MS is donor-derived and guide target therapeutic strategies.

## Supplementary Information

**Additional file 1: Supplementary table 1**: Next generation sequencing (NGS) assay of 34 commonly mutated genes in AML/MPN/MDS.

## Data Availability

All data and material were presented in this published article.

## Abbreviations

PTLD: Post-transplant lymphoproliferative disorders
MS: Myeloid sarcoma
NGS: Next-generation sequencing
sCr: Serum creatinine
APL: Acute promyelocytic leukemia
AML: Acute myelocytic leukemia
MPN: Myeloproliferative neoplasm
MDS: Myelodysplastic syndrome
ATRA: All-trans retinoic acid; ATO: arsenic trioxide;
