# Supplementary material for: Case report: Rare myeloid sarcoma development following renal transplantation with KRAS and DNMT3A gene mutations
Source: Diagn Pathol. 2021 Aug 31;16:82. doi: 10.1186/s13000-021-01141-z (PMC8406562; doi:10.1186/s13000-021-01141-z)
Supplement: Supplementary file 1 — Additional file 1: Supplementary table 1: Next generation sequencing (NGS) assay of 34 commonly mutated genes in AML/MPN/MDS. [file 13000_2021_1141_MOESM1_ESM.docx]

Supplementary table 1: Next generation sequencing (NGS) assay of 34 commonly mutated genes in AML/MPN/MDS

| *BCOR* | *ASXL1* | *BCORL1* | *CARL* | *CBL* | *CEBPA* | *CSF3R* | *DNMT3A* | *ETV6* |
| --- | --- | --- | --- | --- | --- | --- | --- | --- |
| *FLT3* | *GATA2* | *IDH1* | *IDH2* | *JAK2* | *KIT* | *KRAS* | *MLL* | *MPL* |
| *NRAS* | *PDGFRA* | *PHF6* | *PIGA* | *RUNX1* | *SETBP1* | *SF3B1* | *SH2B3* | *SRSF2* |
| *TP53* | *U2AF1* | *WT1* | *ZRSR2* | *EZH2* | *NPM1* | *TET2* |  |  |
